# The Role of Polymorphisms in Collagen-Encoding Genes in Intervertebral Disc Degeneration

**DOI:** 10.3390/biom11091279

**Published:** 2021-08-26

**Authors:** Vera V. Trefilova, Natalia A. Shnayder, Marina M. Petrova, Daria S. Kaskaeva, Olga V. Tutynina, Kirill V. Petrov, Tatiana E. Popova, Olga V. Balberova, German V. Medvedev, Regina F. Nasyrova

**Affiliations:** 1 The Neurological Department No. 16, The Hospital for War Veterans, 193079 Saint-Petersburg, Russia; 2 The Center of Personalized Psychiatry and Neurology, V. M. Bekhterev National Medical Research Center of Psychiatry and Neurology, 192019 Saint-Petersburg, Russia; 3 The Center for Collective Usage “Molecular and Cell Technologies”, V. F. Voino-Yasenetsky Krasnoyarsk State Medical University, 660022 Krasnoyarsk, Russia; stk99@yandex.ru (M.M.P.); dashakas.ru@mail.ru (D.S.K.); tutynina_lelya@mail.ru (O.V.T.); kllpetrov@mail.ru (K.V.P.); 4 The Department for Epidemiology of Non-Infectious Diseases, The Yakutsk Scientific Center for Complex Medical Problems, 677000 Yakutsk, Russia; tata2504@yandex.ru; 5 The Olympic Sports Research Institute, The Ural State University of Physical Culture, 454091 Chelyabinsk, Russia; olga-balberova@mail.ru; 6 The Department of Hand Surgery with Microsurgical Equipment, R. R. Vreden National Medical Research Center for Traumatology and Orthopedics, 195427 Saint-Petersburg, Russia; dr.medvedev.g@yandex.ru

**Keywords:** intervertebral disc, degeneration, candidate genes, genetics, genetic predisposition, back pain, collagen, intervertebral disc disease

## Abstract

(1) Background: The purpose of this review is to analyze domestic and foreign studies on the role of collagen-encoding genes polymorphism in the development of intervertebral discs (IVDs) degeneration in humans. (2) Methods: We have carried out a search for full-text articles published in e-Library, PubMed, Oxford Press, Clinical Case, Springer, Elsevier and Google Scholar databases. The search was carried out using keywords and their combinations. The search depth was 5 years (2016–2021). In addition, this review includes articles of historical interest. Despite an extensive search, it is possible that we might have missed some studies published in recent years. (3) Results: According to the data of genome-wide and associative genetic studies, the following candidate genes that play a role in the biology of IVDs and the genetic basis of the processes of collagen degeneration of the annulus fibrosus and nucleus pulposus of IVDs in humans are of the greatest interest to researchers: *COL1A1*, *COL2A1*, *COL9A2*, *COL9A3*, *COL11A1* and *COL11A2.* In addition, the role of genes *COL1A2*, *COL9A1* and others is being actively studied. (4) Conclusions: In our review, we summarized and systematized the available information on the role of genetic factors in IVD collagen fibers turnover and also focused on the functions of different types of collagen present in the IVD. Understanding the etiology of impaired collagen formation can allow doctors to prescribe pathogenetically-based treatment, achieving the most effective results.

## 1. Introduction

**Figure 1:**
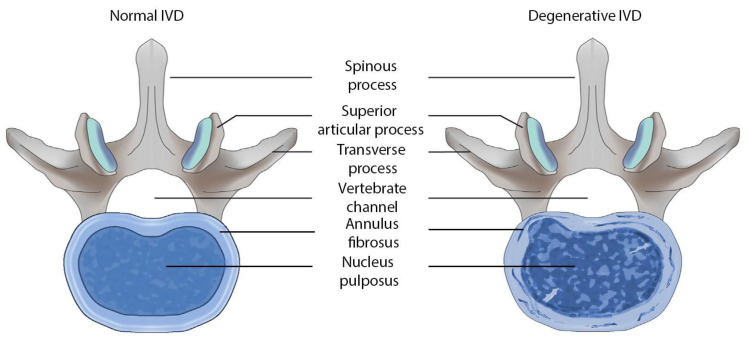
Schematic representation of the human lumbar intervertebral disc (IVD) in normal conditions and with its degeneration.

Intervertebral disc degeneration (IVDD) (Figure 1) is a common pathology of the spine that significantly reduces the quality of life and performance of patients [1]. Neurologists, therapists, rheumatologists and orthopedists take part in the observation and treatment this disorder. Chronic pain syndromes associated with IVDD lead to significant economic losses [2,3]. About 80–90% of patients with acute low back pain (LBP) have persistent regression of pain syndrome after conservative treatment, but the remaining 10–20% develop chronic pain syndrome [4]. The pain chronicity leads to an increase in the temporary disability periods in patients and sometimes to their disability [5].

**Figure 2:**
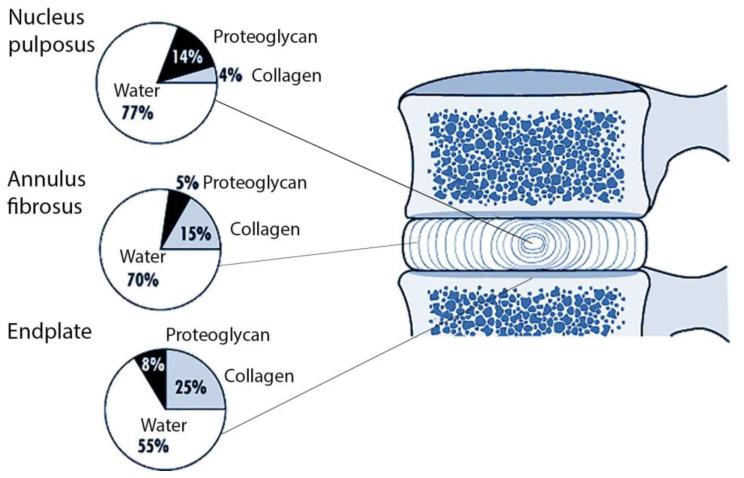
The relative proportions of the three main components of the normal adult intervertebral disc and the endplate.

The etiology of IVDD as a multifactorial disease includes a genetic predisposition and exposure to environmental factors [6]. Over the past decade, it has been convincingly shown that the role of genetic predisposition is dominant. Some of the genetic factors of IVDD have already been identified, but most of them are not known [7,8,9,10]. However, the genetic mechanisms of IVDD are currently poorly understood. This review is focused on the analysis of studies on the collagen as a part of the intervertebral disk (IVD) (Figure 2) and its dysfunction and degeneration genetic basis. This is one of the mechanisms of IVDD-development.

The genetic aspects of turnover (synthesis, functioning and degradation) of collagen fibers and their role in health and disease are under active study. The largest number of works is devoted to collagen of bone tissue and internal organs. The number of studies concerning genetic predictors of collagen formation in the IVDs has increased in recent years, but there is a need to systematize the existing data.

The purpose of this review is to analyze domestic and foreign studies on the role of collagen-encoding genes polymorphism in the development of intervertebral discs (IVDs) degeneration in humans.

## 2. Materials and Methods

We have carried out a search for Russian-language and English-language full-text articles published in e-Library, PubMed, Oxford Press, Clinical Case, Springer, Elsevier and Google Scholar databases. The search was carried out using keywords and their combinations, including intervertebral disc, degeneration, candidate genes, genetics, genetic predisposition, back pain, collagen and intervertebral disc disease.

The search depth was 5 years (2016–2021). In addition, this review includes articles of historical interest. Despite an extensive search, it is possible that we might have missed some studies published in recent years.

We analyzed 324 publications, of which 38 met the objectives of this review, including 20 associative genetic studies of SNVs of candidate genes predisposing to IVDD development: *COL1A1*, *COL1A2*, *COL2A1*, *COL9A1*, *COL9A2*, *COL9A3*, *COL11A1* and *COL11A2.*

## 3. Results

One of the most important functions of the IVDs, protection from mechanical damage, is realized due to the mechanism of reversible deformation of the structure and is possible to a large extent due to collagen fibers. There are ethnic features of the structure of collagen fibers and the cellular composition of the IVDs, but with age, the IVDs becomes thinner, more rigid, less tense and less elastic. Changes also occur in the structure of collagen fibers. Specifically, with age, collagen fibers become more disorganized; the diameter of collagen fibers decreases in people up to 45 years old, while in people after 45 years old, the collagen fiber becomes coarser and more rigid [11].

Collagens play a key role in the regulation of cell migration and differentiation; it has a signaling function since proteins of the cell surface bind to it [12]. Collagen’s interaction with cell surface proteins can be carried out through receptors that recognize amino acid sequences on the collagen molecule. In addition, some proteins can bind to both collagen and integrins, promoting cell adhesion and proliferation [13,14]. When collagen fibers disintegrate, peptide regulatory factors are released that affect further regeneration [15].

Collagens are present in all anatomical structures of the spine, including: IVDs; intervertebral joints; ligaments, tendons and muscles; membranes (dura mater, arachnoid, pia mater)-protective layers that surround and nourish the spinal cord; and blood vessels [16,17].

In humans, like in other vertebrates, 28 types of the collagen are currently identified (additionally, there is collagen XXIX which belongs to the class of collagens and contains von Willebrand’ factor type A), encoded by at least 45 different genes [18]. The composition of collagen fibers varies in different organs depending on the functions of the corresponding organ [19]. The IVD, which bears mechanical stress, is dominated by fibrillar collagens (including a large amount of type I collagen). Currently, several collagen types that are part of the IVDs have been studied. Collagens of types I and II are the main components of the IVDs. The peripheral extracellular matrix of the annulus fibrosus contains mainly collagen type I with a relatively low content of proteoglycan and water. The inner part of the annulus fibrosus contains more collagen type II and proteoglycans. Typically, collagens make up about 60% of the dry weight of the annulus fibrosus, while proteoglycans make up about 25%. Other collagen types, such as collagen types XI and IX, which play a role in the assembly of collagen type II fibers and the formation of cross-links between adjacent collagen fibrils, constitute a small part of the extracellular matrix. Compared to the annulus fibrosus, the extracellular matrix of the nucleus pulposus contains a large amount of collagen type II and proteoglycans [20].

**Figure 3:**
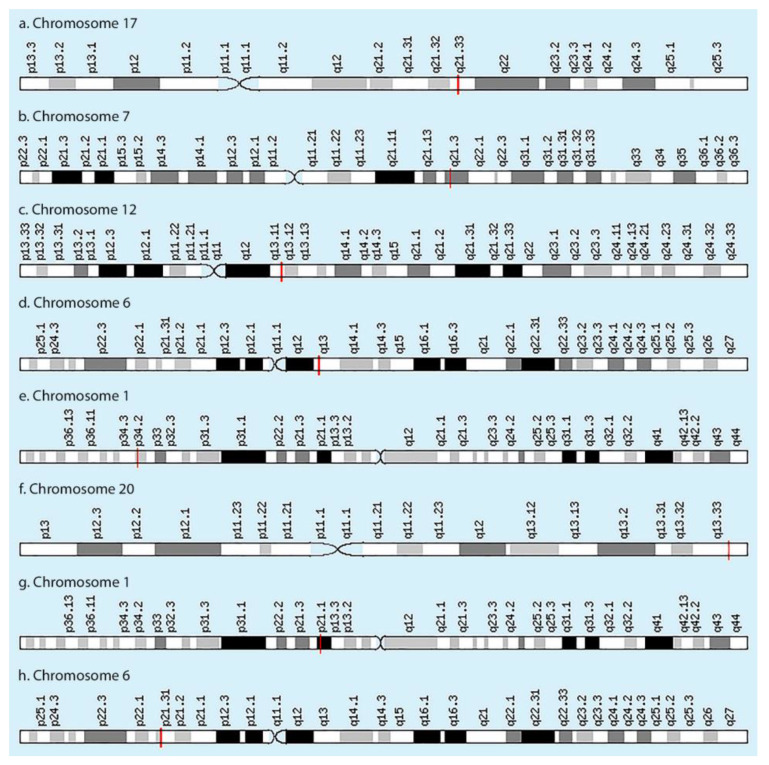
Chromosomal localization of the collagen-encoding genes: (**a**) *COL1A1*; (**b**) *COL1A2*; (**c**) *COL2A1*; (**d**) *COL9A1*; (**e**) *COL9A2*; (**f**) *COL9A3*; (**g**) *COL11A1*; (**h**) *COL11A2*.

There are two groups of factors that can influence the synthesis of collagen in the IVDs: external and internal ones. External factors include the type of the diet (the completeness of the intake of nutrients necessary for collagen synthesis) and the impact of environmental factors. Internal factors include the state of the hormonal background; the genetic code of the structural elements of the IVD, inherent at birth; epigenetic regulation of the activity of genes encoding key proteins and enzymes of collagen formation. Thus, the role of collagens in the mechanical and elastic functions of IVDs is great, which explains the growing interest of researchers and clinicians in studying the predictive role of single nucleotide variants (SNVs) of genes, encoding collagen types I, II, IX and XI in the development of IVDD: *COL1A1*, *COL1A2*, *COL2A1*, *COL9A1*, *COL9A2*, *COL9A3*, *COL11A1* and *COL11A2* (Figure 3). 

Thus, monogenic diseases caused by mutations of *COL1A1*, *COL1A2*, *COL2A1*, *COL9A1*, *COL9A2*, *COL9A3*, *COL11A1* and *COL11A2* genes [21] (Table 1) and SNVs of these genes may be important in the IVDD development. 

### 3.1. COL1A1 Gene

The *COL1A1* gene located at chromosomal locus 17q21.33 and is 18 kb (kilo bases) in size and is composed of 52 exons. This gene encodes the pro-alpha1 chains of type I collagen whose triple helix comprises two alpha1 chains and one alpha2 chain. Type I collagen is a fibril-forming collagen found in most connective tissues and is abundant in bone, cornea, dermis and tendon. Mutations in this gene are associated with osteogenesis imperfecta types I-IV, Ehlers–Danlos syndrome type VIIA, Ehlers–Danlos syndrome Classical type, Caffey disease and idiopathic osteoporosis. Reciprocal translocations between chromosomes 17 and 22, where this gene and the gene for platelet-derived growth factor beta are located, are associated with a particular type of skin tumor called dermatofibrosarcoma protuberans, resulting from unregulated expression of the growth factor (Table 1). The *COL1A1* gene is considered as a candidate gene for IVDD, because the gene is expressed both in and in the annulus fibrosus (primarily) and the nucleus pulposus (secondarily) of the IVD and collagen I alpha 1 is a major component of the fibrous structure of annulus fibrosis in IVDs [22].

The collagen type I is a fibrillar type collagen and most likely the best investigated collagen. This collagen is the most abundant collagen and is the key structural composition of several tissues. It is expressed in almost all connective tissues and the predominant component of the interstitial membrane. The *COL1A1* gene mutations have documented important roles in a range of diseases, with particular focus on bone and connective tissue diseases, in particular osteogenesis imperfecta and Ehlers–Danlos syndrome (Table 1). In addition, the collagen type I is predominantly modified at the posttranslational level, with several crosslinks and other modifications. Several biomarkers of collagen type I have been developed, of both collagen type I degradation and formation, as surrogate makers of bone and IVD degradation and formation, respectively. The collagen type I formation is also associated with fibrosis and fibrogenesis [23].

The collagen type I in a glycosaminoglycan matrix induces proteoglycans synthesis by canine IVD cells [24]. In mice genetically engineered for reduced type I collagen, IVD tissue was also mechanically inferior when compared with control animals [25]. It is therefore plausible that an increased ratio of *COL1A1* expression compared with *COL1A2* may lead to structural alterations, as well as to healing defects in the annulus fibrosus and other components of the discs in IVDD.

Although the mechanism by which genetic changes in type I collagen affect the development of IVDD is not fully understood, various population studies have reported that the rs1800012 polymorphism of the *COL1A1* gene is associated with an increased risk of IVDD. The researchers suggested that this SNV leads to an imbalance between the expression of the *COL1A1* and *COL1A2* proteins, which causes instability of collagen fibers [26]. In Sp1 polymorphism (rs1800012), the guanine (G) is substituted by thymidine (T) in the fourth Sp1 binding site in intron 1 of COL1A1, more specifically, in the promoter +1245 base pair (bp) from the transcription start site [27].

In a study on the Netherlands population, it was shown that individuals over 65 years old who are homozygous carriers of the TT rs1800012 genotype had 3.6 times higher susceptibility to IVDD than people with the GT or GG genotypes [28].

Later, the frequency of carriage of alleles of the rs1800012 polymorphism in young men in Greece was investigated. Comparable results are shown: carriers of the homozygous TT genotype accounted for 33.3% among patients with IVDD, but this genotype was not found in the control group. In addition, a significantly smaller number of controls was heterozygotes for this allele: 66.7% in the IVDD patients v 41.7% in the controls [29]. Elderly men and women with the TT genotype had a higher risk of IVDD than those with the GG and GT genotypes, with an odds ratio (OR) of 3.6. However, in contrast to the study conducted in the Netherlands, there was a high frequency of heterozygous carriers (66.7%) of the GT genotype in the main group (IVDD patients) compared with the healthy control group (41.7%) [28,29].

A study in Finland investigated the predictive role of rs2075555 in the development of IVDD in the lumbar spine. A statistically significant association of the studied polymorphism with the development of degenerative changes in the lumbar IVD in Finns was found [30].

A study in India showed that the rs1800012 polymorphism does not appear to be associated with IVDD in this population. Nevertheless, the frequency of carriage of the minor T allele was higher in patients with IVDD at the cervical and lumbar spine levels compared with the control group, but the differences did not reach statistical significance. The authors suggested that either another polymorphism of the *COL1A1* gene or a polymorphism of the gene encoding a different type of collagen may play a more important role in the development of IVDD in the Indian population [26].

In 2017, Zhong et al. conducted a meta-analysis of studies on the role of rs1800012 polymorphism as a predictor of IVDD among Chinese people. A statistically significant association of homozygous carriage of the minor T allele of this polymorphism with the development of IVDD, including severe forms, has been demonstrated [31].

In 2020, Hanaei et al. studied two groups of patients with IVDD in the Iranian population, comparable in age and sex, in order to study the association of SNV rs909102 of the *COL1A1* gene. The results showed that the T allele, genotypes CC and TT rs909102 SNV of the *COL1A1* gene were more common among patients with IVDD; however, the statistical data turned out to be insignificant [32].

In summary, we can conclude that *COL1A1* is a candidate gene associated with the pathogenesis of IVDD in humans. However, the predictive role of the studied SNVs depends on the region of residence, race and ethnicity of the patients.

### 3.2. COL1A2 Gene

The *COL1A2* gene is located at chromosomal locus 7q21.3. This gene encodes the pro-alpha2 chain of type I collagen [33]. The gene is expressed both in and in the annulus fibrosus (primarily) and the nucleus pulposus (secondarily) of the IVD [34,35].

Mutations in this gene are associated with osteogenesis imperfecta types I-IV, Ehlers–Danlos syndrome type VIIB, recessive Ehlers–Danlos syndrome Classical type, idiopathic osteoporosis and atypical Marfan syndrome. Symptoms associated with mutations in this gene, however, tend to be less severe than mutations in the gene for the alpha1 chain of type I collagen (COL1A1) reflecting the different role of alpha2 chains in matrix integrity [36] (Table 1) and the role of SNVs of this gene in the development of IVDD is doubtful and insufficiently studied. This may be due to the lower expression of the *COL1A2* gene compared to *COL1A1* in IVD structures [37,38].

An enhancer region in the type I collagen alpha 2 chain (pro-COL1A2) promoter has been previously identified approximately −17 kb away from the transcription start site. The study by Ponticos et al. (2004) suggest that the enhancer is central to the activation of the collagen type I and that mice harboring this enhancer/reporter provide a useful model to follow collagen gene transcription activity and for investigating cellular activity in tissue fibrosis and IVDD [39].

### 3.3. COL2A1 Gene

The *COL2A1* gene is located at the 12q13.11 locus and encodes the alpha-1 chain of collagen type II, the main collagen in cartilage. The *COL2A1* gene is considered as a candidate gene for IVDD, because the collagen network of the IVD is formed mostly by collagen type II, along with collagen type I [22] and type IX [40].

The collagen type II is a fibrillar collagen and the main component of cartilage. This collagen is the cartilage collagen; it constitutes 95% of the collagens and approximately 60% of dry weight. The *COL2A1* gene mutations result in several types of chondrodysplasia, leading to premature osteoarthritis. The collagen type II is typically co-assembled with collagen type XI, where it is covalently crosslinked to collagen type IX and interacts with small leucine-rich proteoglycans. Its stability and strength provide the tissue with integrity and resiliency to stress. The collagen type II cleavage is primarily mediated by collagenases of the matrix metalloproteinases (MMPs) family, resulting in well-described biomarkers such as C-terminal telopeptide of collagen type II and C2C. In addition, several formation makers have been developed. The collagen type II has important binding partners such as fibronectin and other collagens [41].

In 2016 and 2017, two similar associative genetic studies were carried out. The first study showed that the SNVs rs2276454 and rs2070739 of this gene are predisposing factors for the development of IVDD [42]. A second case-control study was conducted to assess the association of three *COL2A1* SNVs (rs1793953, rs2276454 and rs1793937) with the risk of IVDD in the Chinese Han population. The results showed that the rs2276454 and rs1793953 variants are associated with an increased and decreased risk of developing IVDD, respectively [43]. We can conclude that there is a confirmed link between rs2276454 and a predisposition to IVDD.

### 3.4. COL9A1 Gene

Type IX collagen belongs to the group of fibril-associated collagens (FACITs) and is a component of collagen fibrils in hyaline cartilage. FACITs modulate the surface properties of the fibrillar collagens by incorporating in the interfibrillar space in staggered fibrils. It forms a heterotrimer with three different chains, α1(IX)α2(IX)α3(IX), encoded by genes *COL9A1* (chromosomal locus 6q13), *COL9A2* and *COL9A3* [44], respectively. Mutations in *COL9A1*, *COL9A2* and *COL9A3* cause multiple epiphyseal dysplasia (MED), an autosomal dominant chondrodysplasia, Stickler syndrome (Table 1).

The domains of type IX collagen are three triple helical domains (COL1, COL2 and COL3) separated and flanked by nontriple helical (NC) domains, NC1–NC4. There is a splicing variant in the NC4 domain of the α1(IX) chain. The NC3 domain has one chondroitin sulfate chain attachment site. The length of the chondroitin sulfate chain may vary depending on the tissue type and developmental stage. The short form of type IX collagen in chick embryo vitreous humor has a long chondroitin sulfate chain in the NC3 domain of the α2(IX) chain.280 The α1(IX) chain in bovine nucleus pulposus, the central zone of the IVD, has only a short form of the α1(IX) chain and lacks the NC4 domain, while the α1(IX) chain in hyaline cartilage has the long form of the α1(IX) chain. Type IX collagen molecules assemble with type II and XI collagens to form the fibrils in hyaline cartilage [45].

So, this collagen is a fibril-associated collagen with interrupted triple helices. The collagen type IX is present in the chondrocytes of growth-plate cartilage, adult articular, cartilage and IVDs. Mutations in type IX collagen can predispose individuals to multiple epiphyseal dysplasia, a clinically highly heterogeneous skeletal disorder, with early-onset osteoarthritis as a very common manifestation. In addition, type IX collagen contributes to the stabilization of the fibrillar collagen network in the cartilage matrix and the anchorage of matrilin 3 and proteoglycans, which controls the diameter of collagen fibrils; consequently, it is an essential part of articular cartilage [46]. Both the nucleus pulposus and the annulus fibrosus of the IVD contain small amounts of type IX collagen, which is believed to serve as a bridge between collagens and non-collagen proteins in tissues [7].

Transgenic mice expressing mutant α1 (IX) collagen develop IVDD and progressive joint degeneration with age [47]; at 20 months, these mice show protrusion of the disc with impingement on the spinal cord, mimicking human herniation [48]. Bächinger et al. (2010) demonstrated, that the mouse model with inactivated *COL9A1* gene does not synthesize type IX collagen. *COL9A1* knockout mice develop normally without detectable abnormalities, but severe joint disease develops by approximately 9 months of age [45].

So, more than 20 years ago, it was found that transgenic mice overexpressing the mutant *COL9A1* gene and mice with inactivated *COL9A1* gene exhibited accelerated IVDD and more pronounced IVD hernia than in the control group of animals of the same age (with correct expression of *COL9A1*) [49].

Nucleus pulposus, the central zone of the IVD, is gel-like and has a similar collagen phenotype to that of hyaline cartilage. The sequence encoded by exon 7, predicted from human *COL9A1*, is absent from both short and long forms of alpha1(IX) from bovine nucleus pulposus and articular cartilage. A structural analysis of the cross-linking sites occupied in type IX collagen from nucleus pulposus showed that usage of the short alpha1(IX) transcript in disc tissue had no apparent effect on cross-linking behavior. As in cartilage, type IX collagen of nucleus pulposus was heavily cross-linked to type II collagen and to other molecules of type IX collagen with a similar site occupancy [50].

However, in studies involving people with IVDD, more attention has recently been paid to two other genes. This is due to those that type IX collagen is the short molecular form that lacks a non-collagenous (NC)4 domain, not the long form found in most hyaline cartilages. Protein sequence and reverse transcriptase-PCR analysis confirmed this was the result of expression from the alternative transcription start site, not proteolysis of the long form. In view of recent reports that common SNVs in *COL9A2* and *COL9A3* genes are linked to chronic sciatica associated with IVD pathology, the specific interactions and role of *COL9A2* and *COL9A3* genes in IVDD are important to define [51].

### 3.5. COL9A2 Gene

The *COL9A2* gene is located at chromosomal locus 1p34.2. This gene encodes one of the three alpha chains of type IX collagen, the major collagen component of hyaline cartilage. Type IX collagen, a heterotrimeric molecule, is usually found in tissues containing type II collagen, a fibrillar collagen. This chain is unusual in that, unlike the other two type IX alpha chains, it contains a covalently attached glycosaminoglycan side chain. Mutations in this gene are associated with multiple epiphyseal dysplasia, Stickler syndrome type V (Table 1). In mice, abnormal type COL9A2 causes IVDD and the skeletal phenotypes of collagen IX-deficient mice suggest that this protein is essential for the functional longevity of the IVD [48]. In a recent study, it was found that collagen type IX and not the collagen I was associated with IVDD by Mayer et al. [40]. A similar association between collagen IX polymorphism and IVDD was reported in Indian patients earlier by Rathod et al. [52].

The Trp2 allele, also known as c.976_977delCAinsTG, p.Gln326Trp and Q326W (rs137853213), is a rare allele of the *COL9A2* gene that results in the replacement of the amino acid arginine with tryptophan in the alpha2-chain protein of type IX collagen and its relationship with IVDD was studied more than 20 years ago [53]. Such mutations result in changes in the structure of the IVD, such as annular ruptures and hernias of the end plate with an odds ratio of 2.4 and 4.0, respectively. Inheritance of the Trp2 allele is associated with IVD diseases in the families of all patients [7,49]. So, a subsequent study of Finnish families revealed that family members who carry the Trp2 allele have a greater degree of IVDD and end-plate [54]. However, the Trp2 association was not replicated in a German study of 250 surgically treated patients who experienced disc prolapse of the cervical and lumbar spine [55]. Seki et al. (2006) examined the association of the *COL9A2* gene SNVs with IVDD in the Japanese population. The authors identified a total of 43 *COL9A2* polymorphisms in addition to Trp2. Of these, they selected and genotyped nine SNVs, including Trp2, which were in the coding region or had minor allele frequencies greater than 20%. One SNV (−2066C > A) showed a positive association (*p* < 0.05), but after Bonferroni’s correction for multiple testing, no SNV showed significant association with IVDD. In contrast, with the Finnish population, Trp2 is common in Japanese individuals and does not associate with IVDD. However, the authors have identified a haplotype of *COL9A2* that associates specifically with severe IVDD. Three SNVs (−2066C > A, c.977A > G and IVS19 + 1147G > C) were selected for the haplotype study by SNV tagger. Through this analysis, the authors found that the “221” haplotype was over-represented in IVDD (*p* = 0.025). Further, this association was more significant when tested for the group with severe (Schneiderman’s grade 10–20) IVDD (*p* = 0.011). The Trp2 allele was linked to the “111” haplotype rather than to the “221” haplotype. [56].

However, according to recent studies, the distribution of the alleles and genotypes of the SNV rs137853213 of the *COL9A2* gene was not reliably associated with IVDD in the Iranian population [32].

### 3.6. COL9A3 Gene

The *COL9A3* gene is located at chromosomal locus 20q13.33 and encodes the alpha 3 chain of type IX collagen. COL9A3, an extracellular matrix molecule is present in the nucleus pulposus of the IVDs and cartilage [57]. Mutations in the *COL9A3* gene could cause chondrodysplasias in humans as well as articular cartilage and IVDD in mice [48]. The *COL9A3* gene was observed to be a key genetic influencer in the process of IVDD [58]. Previous studies have reported the association between the *COL9A3* Trp3 polymorphism and IVDD, but with conflicting results.

The Trp3 mutation, also known as c.307C > T, p.Arg103Trp and R103W (rs61734651), which results in the Arg103Trp amino acid substitution in the third chain of type IX collagen, increases the risk of IVDD. A study in Finland showed that the number of Trp3 allele carriers (24%) among patients with IVDD was statistically significantly higher compared to healthy people in the control group (9%) [44]. According to another study conducted in Poland, an association was also found between the Trp3 allele and the development of spinal pathology: the Arg103Trp substitution in the amino acid sequence of the alpha-3 chain of collagen IX was found in 12.3% of patients with IVDD compared to 4.7% among healthy people from the control group [49].

Wu et al. in 2018 published a meta-analysis of studies examining the association between the carriage for the SNVs of the *COL9A2* and *COL9A3* genes and a predisposition to IVDD. The meta-analysis included 10 case-control studies, including 2102 cases of IVDD and 2507 controls and showed that SNVs of the *COL9A2* gene (rs12077871, rs12722877 and rs7533552) and *COL9A3* gene (rs61734651) were not significantly associated with IVDD. Egger’s test and Begg’s funnel graph showed no evidence of publication bias. From this it can be concluded that larger and better designed studies are needed to confirm this hypothesis [59].

With the studies with larger sample sizes of predisposing gene SNVs, it would be much more reliable to discover the connection between candidate genes and IVDD. In order to solve the inconsistence, meta-analysis by Huang et al. (2018) [44] was performed to examine the association of *COL9A3* Trp3 polymorphism with IVDD risk by critically reviewing 11 studies. Its strength came from the accumulation of various published data, offering more information to explore significant differences. This study included research of Asia (Iran, China and India), Europe (Finland, Greece and Turkey) and America (USA), containing different kinds of ethnicities and enrolling both male and female. Therefore, the results are much more comprehensive. Moreover, several strategies and strict principle were applied to evaluate the methodological quality of the studies and most of the studies included in this meta-analysis possessed moderate or high qualities. Limited to data, the authors only analyzed trp3 positive versus trp3 negative to estimate the ORs and 95% CI rather than five models (allele, homozygote, recessive, dominant and heterozygote models). Basing on the epidemiological evidence, the meta-analysis suggested that the *COL9A3* gene Trp3 polymorphism did not seem to be connected to risk of IVDD in any gender, continent or ethnicity of people [44]. Future research with larger sample sizes is required to verify this outcome.

### 3.7. COL11A1 Gene

The *COL11A1* gene (chromosomal locus 1p21.1) and the *COL11A2* gene, encode one of the two alpha chains of type XI collagen, an extracellular matrix protein specific for cartilage tissue, used in the organization of the extracellular matrix and the formation of cartilage-collagen IVD fibrils. These chains fold into triple helical heterotrimers (the third alpha-chain of type XI collagen is a post-translationally modified alpha-1 chain of type II collagen) to form procollagen, which is secreted in the extracellular matrix, where it participates in the formation of fibrils with other specific collagens and regulates the diameter collagen fibrils of cartilage [7,49].

Type XI collagen is a fibrillary collagen. This collagen is broadly distributed in articular cartilage, tendons, trabecular bone, skeletal muscle and other tissue (testis, trachea, placenta, lung and the neoepithelium of the brain). Type XI collagen is able to regulate fibrillogenesis by maintaining the spacing and diameter of type II collagen fibrils and a nucleator for the fibrillogenesis of collagen types I and II. The *COL11A1* gene mutations in type XI collagen are associated with Stickler syndrome, Marshall syndrome, fibrochondrogenesis (Table 1) [21]. Type XI collagen binds heparin, heparan sulfate and dermatan sulfate. Currently, there are no biomarkers for type XI collagen [46]. Type XI collagen is a minor component of cartilage collagen fibrils, but it is present in the annulus fibrosus and nucleus pulposus of IVDs [60]. The structural integrity of extracellular matrix and the physiologic balance of its components are critical to IVDs function. Perturbation of extracellular matrix metabolism would increase the mechanical load of the IVDs, leading to IVDD. The reduction in type XI collagen, the critical organizer of extracellular matrix, ultimately causes disintegration of extracellular matrix and, hence, IVDD [36].

Due to the interaction with collagen types II and IX in the IVD, collagen type XI and its coding genes have been studied as possible factors contributing to the development of IVD diseases and some studies have shown the association between polymorphism c.4603C > T of *COL11A1* gene and IVDD. The frequency of the 4603T allele was significantly higher in the patients with IVDD than in the healthy controls [7,33,60].

Mio et al. (2007) first examined the association of the type XI collagen genes (*COL11A1*, *COL11A2* and *COL2A1*) with IVDD, which included patients with and without lumbar disc herniation (LDH). To gain insight into the role of type XI collagen in LDH, the authors examined *COL11A1* expression in tissues and cells by quantitative real-time PCR. *COL11A1* mRNA was predominantly expressed in IVD. In addition, they investigated the correlation between the *COL11A1* mRNA expression level and a variety of LDH phenotypes and found that severity of IVDD evaluated by MRI was inversely correlated with *COL11A1* expression in IVDs of patients with LDH. The authors analyzed the expression and localization of type XI collagen in IVD by immunohistochemistry. Normal discs had a highly uniform extracellular matrix structure, with intense immunostaining of type XI collagen in the nucleus pulposus cells and extracellular matrix. In degenerative discs, however, weak immunostaining of type XI collagen around the nucleus pulposus cells were observed. These findings implicate a decrease of type XI collagen in the pathogenesis of LDH. A statistically significant association was found between the SNV rs1676486 (c.4603C→T) of the *COL11A1* gene and hernia of the lumbar IVD in Japanese patients with lower back pain. The expression level of the susceptibility allele c.4603T was significantly lower than that of the c.4603C allele. This SNV may affect *COL11A1* transcription by altering mRNA stability and examined the stability of *COL11A1* mRNA containing the SNV. The transcript containing the susceptible allele degraded faster. In addition, a decrease in the expression of the *COL11A1* gene was associated with a hernia of the lumbar IVD and a higher degree of its degeneration [61].

By Liu et al. in 2017, a similar study was conducted aimed at studying the relationship of this genetic variant of *COL11A1* with the development of IVD hernia in the lumbar spine in representatives of the Chinese population. SNV rs1676486 *COL11A1* was genotyped in Chinese with IVD hernia and healthy controls. As a result of the study, it was found that patients had a significantly higher frequency of carriage of the homozygous TT genotype than the control group (10.2% versus 7.3%, respectively). In addition, the frequency of carriage of the T allele was significantly higher in patients with IVD hernia than in the control group (34.8% versus 28.1%, respectively). Patients with the TT genotype were found to have significantly more severe IVDD. In addition, the expression of the *COL11A1* gene in the lumbar disc was significantly lower in patients with the TT genotype than in patients with the CT or CC genotype. Moreover, the level of expression was inversely correlated with the severity of IVDD. Thus, the association between rs1676486 and IVDD was confirmed in the Chinese population [62]. Whether there is a predictive role for this SNV in European populations and what role it plays in the IVDD process remains to be studied.

Jiang et al. (2017) observed 428 patients with IVDD and 400 normal controls. This study was conducted by examining two aspects: environmental factors and SNVs genotyping. The environmental factors were evaluated with a questionnaire survey including questions about body mass index, smoking habits, the physical demands of their job and exposure to vibrations. Rs1337185, rs5275, rs5277, rs7575934, rs3213718 and rs162509 were genotyped using a PCR-based invader assay. The genotype and allele frequencies of rs1337185 and rs162509 were significantly different between the patients with IVDD and the normal controls. In rs1337185, a significant association was found between the C allele (risk allele) and the presence of IVD herniation (OR = 1.80; 95% CI 1.21 to 2.68; *p* = 0.003, adjusted *p* = 0.012). In rs162509, the G allele represented 1.58-fold increased risk to suffer from disc herniation (OR = 1.58; 95% CI 1.20 to 2.09; *p* = 0.001, adjusted *p* = 0.004). The authors were shown that the SNPs rs1337185 in the *COL11A1* gene and rs162509 in the *ADAMTS5* gene are associated with susceptibility to IVDD. The C allele of rs1337185 is risky for patients who are affected by lumbar pathologies such as IVD herniation, stenosis and spondylolisthesis. The G allele of rs16250 represents a risk factor for the development of IVD herniation [63]. These results are consistent with earlier study by Rajasekaran (2015) in a population of young Indians (<40 years) with IVDD [64] including 308 patients with mild Total Disc Degenerative Score (TDDS) and 387patients with severe TDDS were studied. The genetic association analysis for 58 single SNVs of 35 candidate genes was performed including the *COL11A1* gene. This shows that IVDD is a complex disease with an intricate interplay of multiple genetic polymorphisms. These five genes (*COL11A1*, *ADAMTS5*, *CALM1*, *IL1F5* and *COX2*) have different functions in the matrix metabolism, intracellular signaling and inflammatory cascade. Rs1337185 of the *COL11A* gene (*p* = 0.02) is found to be significantly associated with severe TDDS.

### 3.8. COL11A2 Gene

The *COL11A2* gene is located at chromosomal locus 6p21.32 and encodes one of two alpha chains of type XI collagen. Stickler syndrome type III, otospondylomegaepiphyseal dysplasia (OSMED syndrome), Weissen–Bacher–Zweymuller syndrome, autosomal dominant non-syndromic sensorineural type 13 deafness (DFNA13), autosomal recessive non-syndromic senso-rineural type 53 deafness (DFNB53) are associated with loss-of-function mutations in the *COL11A2* gene [21,65] (Table 1).

Type XI collagen, a quantitatively minor component of extracellular matrix, is important for cartilage collagen fibril formation and extracellular organization [36]. Lawrence et al. (2015) showed that in *COL11A2* mutants, type II collagen is made but is prematurely degraded in maturing cartilage and ectopically expressed in the joints. These changes are correlated with increased stiffness of both bone and cartilage; quantified using atomic force microscopy. Taken together, these data demonstrate a key role for type XI collagen in maintaining the properties of cartilage matrix [66]. Moreover, patients with rs9277935 genotype TT have a significantly increased expression of *COL11A2* than those with genotype GG. In addition, COL11A2 demonstrated chondrogenic properties in vitro [67].

Working model by Caldeira et al. (2017) [68] for the changes that occur in the IVD microenvironment with development and ageing is very important. So, the authors are shown that from the fetal stages to adulthood, collagen type XII and XIV expression is lost and only collagen type XI is maintained. With increasing age, there is an enrichment in fibronectin and prolargin. Proteomic alterations are accompanied by matrix remodeling (fibrillogenesis and fibril organization are both affected), concomitant with water loss and a cell population decline. This ultimately causes age-associated IVDD, hernia formation and back pain. In addition, the authors were shown that and *COL11A2* plays a role in this working model of IVDD [68]. This is consistent with the opinion of other authors that the *COL11A2* gene is related to the development of IVDs [69,70]. Noponenhietala et al. (2003) found in rs1800587 of the *COL11A2* gene individual carrying the risk T allele had an increased risk of developing degenerative lumbar spinal stenosis, which may relate to underlying degeneration [69].

It was previously found that variation in the sequence of intron 9 of the *COL11A2* gene is associated with an increased risk of IVDD compared to people without this polymorphism (the magnitude of the IVD bulge forward and backward along the sagittal midline was estimated using magnetic resonance imaging (MRI) as a sign of degeneration). Another large study, conducted with the participation of men in Finland, showed that two SNVs (rs1463035, rs1337185) of the *COL11A1* gene and one of the three studied SNVs (rs2076311) of the *COL11A2* gene were associated with certain disc bulges and signal intensity from them on MRI data. These SNVs may play a role in the production of unstable transcripts of the disease-associated allele. Instability of gene transcription can cause a decrease in collagen function and subsequent degeneration of the IVD [7,30,71].

By Yang et al. (2018), genotyping of patients with lesions of the lumbar spine and a control group in the Chinese Han population was carried out to study the role of this gene in the development of IVDD. Genotyping was performed for six SNVs of the *COL11A2* gene. The strongest associations with degeneration of the lumbar IVD were observed for the rs2071025 polymorphism. Carriers of the A allele had an increased risk of developing the disease compared to carriers of the G allele. An association was found between the allelic variants of SNV rs2071025 and the risk of developing IVDD in both women and men: the carriers of the A allele had an increased risk of IVDD (OR = 1.47, 95% CI = 1.20–1.80, *p* = 0.0002) as compared with the G allele. However, genetic models have shown that carriage of the C allele rs986522 significantly increases the risk of lumbar IVDD in women. In men, no statistically significant association was found between the carriage of alleles and genotypes of SNV rs986522 and the risk of IVDD [72].

## 4. Discussion

The prevalence of IVDD in the general population varies from 36 to 93% depending on age [73,74]. The estimated transition age at which intervertebral discs lose the growth potential and begin degenerating is 13.3 years. The estimated disc degeneration rate is 0.0344/year [75]. Meta-analysis by Brinjikji (2015) demonstrates that MR imaging evidence of disc bulge, degeneration, extrusion, protrusion, Modic 1 changes and spondylolysis are more prevalent in adults 50 years of age or younger with back pain compared with asymptomatic individuals [76]. Although various environmental factors such as smoking, age, gender and mechanical load increase the risk of IVDD, it is hypothesized that up to 74% of the etiology of IVDD is due to heritability [60,77]. IVDD is most common causes of impairment and disability for middle aged and older people: spine stiffness, neck pain and back pain. This explains the importance of searching for modifiable and non-modifiable risk factors for the degenerative process.

Potential causes of the IVDDs include declining nutrition, loss of viable cells, cell senescence, post-translational modification of matrix proteins, accumulation of degraded matrix molecules and fatigue failure of the matrix. The most important of these mechanisms appears to be decreasing nutrition of the central disc that allows accumulation of cell waste products and degraded matrix molecules, impairs cell nutrition and causes a fall in pH levels that further compromises cell function and may cause cell death [73].

IVDD is a complex condition with environmental factors and multiple genes likely acting together to determine an overall degenerative phenotype. IVDs contain an abundant extracellular matrix of proteoglycans and collagens [73,78]. The IVD matrix comprises mainly a fibrillar collagen network that offers tensile strength and aggregating proteoglycans that resist compressive forces. These major components form a mesh suited for containing water molecules, especially in the nucleus. An intact extracellular matrix is essential to normal disc function. The ability of IVD tissue to withstand mechanical forces largely depends on the structural integrity of the matrix and on the physiological balance of collagen, proteoglycan and water content [34].

**Figure 4:**
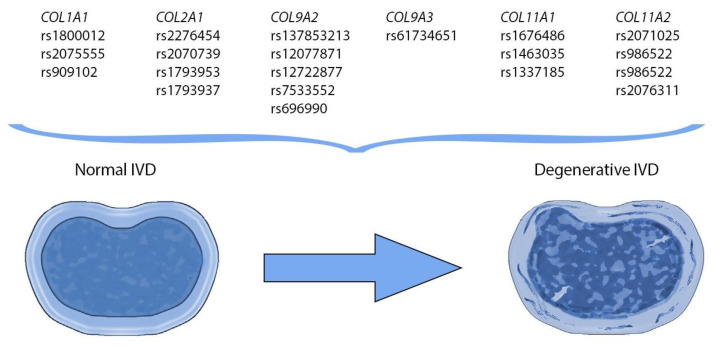
Candidate genes predisposing to intervertebral disc degeneration in humans.

IVDD is believed to begin as early as the second decade of life and is viewed by most as an inevitable consequence of ageing. Despite its prevalence, there is no clear distinction between IVDD and normal maturation, nor is it clear why IVDD progresses slowly in some patients, whereas in others more rapid destruction of the IVD can occur. Various risk factors were thought to be associated with IVDD, including environmental, ergonomic and biometric. There is now growing evidence that genetic factors play a decisive predictive role in the development of IVDD (Figure 4) [79].

Many recent studies show the clinical significance of the association of the rs1800012 polymorphism of the *COL1A1* gene with an increased risk of IVDD [26,27,28,29,30,31]. In addition, recently, the SNV rs909102 of the *COL1A1* gene has been studied, but its association with IVDD in the Iranian population has not been reliably proven [32]. At the same time, the role of the studied SNVs of the *COL1A2* gene in the development of IVDD is questionable, which explains the need for additional studies in various racial and ethnic groups of patients.

In 2016 and 2017, two similar associative genetic studies were carried out, the first of which showed that the SNVs rs2276454 and rs2070739 of the *COL2A1* gene are predictors of the development of IVDD in adult patients [42]. The second study also confirmed that the rs2276454 variant is associated with an increased risk of developing IVDD [43].

The role of SNVs of the genes encoding type IX collagen chains (*COL9A1*, *COL9A2* and *COL9A3*) in the development of pathological processes in IVD is also being actively studied at the present time. At the same time, increased attention of researchers has been paid to the genes *COL9A2* and *COL9A3.* The rare Trp2 allele (rs137853213) of the *COL9A2* gene has been well studied. As a result, it was shown that the inheritance of the Trp2 allele is associated with IVDD in the families of all patients [7,49].

However, according to recent studies, the above-mentioned SNV of the *COL9A2* gene did not show its reliable association with IVDD in the Iranian population [32]. In the latest study of Finnish patients, it was shown that the frequency of carriage of the Trp3 allele (rs61734651) in the *COL9A3* gene among patients with IVDD was statistically significantly higher than in healthy people in the control group [49].

Conducted by Wu et al. in 2018, a large meta-analysis of the association between the *COL9A2* and *COL9A3* SNVs showed that the rs12077871, rs12722877 and rs7533552 SNVs of the *COL9A2* gene and the rs61734651 SNV of the *COL9A3* gene were not significantly associated with the development of IVDD. The authors concluded that more large-scale and well-designed studies are needed to confirm this hypothesis [59].

Other authors have found a statistically significant association between SNV rs1676486 of the *COL11A1* gene and hernia of the lumbar IVD in Asians (using the example of the Japanese [7,61] and Chinese [62] populations).

Another large study, conducted with the participation of Finnish men, showed that two SNVs (rs1463035 and rs1337185) of the *COL11A1* gene and one of the three studied SNVs (rs2076311) of the *COL11A2* gene were associated with certain abnormalities of the IVD surface and signal intensity from it according to MRI data. In the same study, the SNV of the genes *COL1A1* (rs2075555) and *COL9A1* (rs696990) also provided reliable evidence of an association with the presence of pathological signals from IVD according to MRI of the spine [7,30,71].

A study by Yang X. et al. in 2018, in the Chinese Han population, not only indicated an association with degeneration of the lumbar IVD in the rs2071025 SNV of the *COL11A2* gene, but also that another SNV of this gene (rs986522) significantly increases the risk of lumbar IVDD in women [72].

Some limitations of our study should be noted (Table 2). First, only publications in English and Russian were searched and publications in other languages were excluded. Secondly, the present work did not set out to conduct a meta-analysis.

The authors undoubtedly see the need to plan and conduct large, randomized studies on the pathology under consideration with the inclusion of representatives of different ethnic and racial groups, different age groups of both sexes (men and women).

With more than 20 unique SNVs of candidate genes associated with IVDD, the molecular changes in the associated collagens or pathology of the IVDs are not yet fully understood. In the coming years, research targeted toward fully understanding the protein changes of extracellular matrix of IVDs due to the already identified SNVs is crucial. If we can fully understand the molecular changes involved in IVDD, then creating targeted therapeutics based on genetic profiling becomes a possibility. With improved understanding of the genetic variants associated with IVDD and rapid genomic analysis available through next-generation genotype sequencing and DNA tests, the possibility of providing effective personalized medicine can become a reality in the future [80,81,82].

## 5. Conclusions

According to genome-wide and associative genetic studies, the following candidate genes that play a role in IVD biology in humans and the genetic basis of collagen degeneration of the annulus fibrosus and nucleus pulposus are of the greatest interest to researchers: *COL1A1*, *COL2A1*, *COL9A2*, *COL9A3*, *COL11A1* and *COL11A2.* In addition, the role of genes *COL1A2*, *COL9A1* and others is being actively studied. This is important for the development of modern methods of drug and non-drug strategies for the prevention and treatment of IVD pathology.

A large number of SNVs of the candidate genes are being actively studied as genetic predictors (biomarkers) of dysfunction and collagen degradation as one of the mechanisms of IVDD in adults. On the one hand, this indicates the relevance of the problem from a scientific and clinical point of view. On the other hand, this indicates that, at present, there are more questions than answers about the genetics of the interdisciplinary spine pathology under consideration.

## Figures and Tables

**Table 1:** Genes responsible for the structure of collagen fibers in intervertebral disk and diseases caused by their mutations [21].

Gene and Protein/Enzyme Encoded by This Gene	Chromosome Localization	Clinical Manifestations of Mutation/Polymorphism
*COL1A1, * encoding the α1 chain of collagen type I	17q21.33, 51 exons	Osteogenesis imperfecta Classic type of Ehlers-Danlos syndrome Caffey disease Idiopathic osteoporosis
*COL1A2,* encoding α2 chain of collagen type I chain	17q21.3, 52 exons	Osteogenesis imperfecta Ehlers-Danlos syndrome type VII B Idiopathic osteoporosis Atypical Marfan syndrome
*COL2A1,* encoding the α1 chain of collagen type II	12q13.11, 57 exons	Achondrogenesis Chondrodysplasia Early onset familial osteoarthritis SED congenita Langer-Saldino achondrogenesis Kniest dysplasia Stickler syndrome type I Spondyloepimetaphyseal dysplasia Strudwick type
*COL9A1,* encoding the α1 chain of collagen type IX	6q13, 43 exons	Osteoarthritis with early onset with multiple epiphyseal dysplasia Chondrodysplasia type Vl Stickler syndrome
*COL9A2,* encoding the α2 chain of collagen type IX	1p34.2, 35 exons	Multiple epiphyseal dysplasia
*COL9A3, * encoding the α3 chain of collagen type IX	20q13.33, 33 exons	Multiple epiphyseal dysplasia type III
*COL11A1, * encoding the α1 chain of collagen type XI	1p21.1, 71 exons	Stickler syndrome type II Marshall syndrome Susceptibility to lumbar disc herniation
*COL11A2, * encoding the α2 chain of collagen type XI	6p21.32, 70 exons	Stickler syndrome type III Otospondylomegaepiphyseal dysplasia (OSMED syndrome) Weissenbacher–Zweymuller syndrome Autosomal dominant non-syndromic sensorineural type 13 deafness (DFNA13) Autosomal recessive non-syndromic sensorineural type 53 deafness (DFNB53)

**Table 2:** Candidate genes encoding collagens and their single nucleotide variants associated with degeneration of intervertebral discs in humans.

Gene, Chromosomal Locus	Single Nucleotide Variants	Protein	Source
*COL1A1* 17q21.33	rs1800012 rs2075555 rs909102	Alpha 1 chain of collagen type I	[7,26,28,29,30,31,32]
*COL1A2* 7q21.3	n/a *	Alpha 2 chain of collagen type I	[33,36]
*COL2A1* 12q13.11	rs2276454 rs2070739 rs1793953 rs1793937	Alpha 1 chain of collagen type II	[1,42,43].
*COL9A1* 6q13	n/a *	Alpha 1 chain of collagen type IX	[7,49]
*COL9A2* 1p34.2	rs137853213 rs12077871 rs12722877 rs7533552 rs696990	Alpha 2 chain of collagen type IX	[7,44,49,53]
*COL9A3* 20q13.33	rs61734651	Alpha 3 chain of collagen type IX	[44,49,59]
*COL11A1* 1p21.1	rs1676486 rs1463035 rs1337185	Alpha 1 chain of collagen type XI	[7,30,49,61,62]
*COL11A2* 6p21.32	rs2071025 rs986522 rs986522 rs2076311	Alpha 2 chain of collagen type XI	[7,30,71,72]

* n/a–not available.
